# Adverse psychological outcomes in Brugada syndrome: About a Tunisian familial pedigree from Sfax

**DOI:** 10.1192/j.eurpsy.2024.1223

**Published:** 2024-08-27

**Authors:** N. Bouayed Abdelmoula, B. Abdelmoula, A. Assel, E. Fadhlaoui, O. Kaabi

**Affiliations:** Genomics of Signalopathies at the service of Precision Medicine - LR23ES07, Medical University of Sfax, Sfax, Tunisia

## Abstract

**Introduction:**

Patients with Brugada Syndrome (BS), a rare inherited cardiac channelopathy, with an increased risk of developing arrhythmias, syncope and sudden cardiac death, also present serious adverse psychological outcomes that require medical support to improve their health and well-being as well as those of their families.

**Objectives:**

Here we report psychological concerns of a Tunisian patient who presented to our genetic counselling, with his three children, for molecular exploration of BS type 1.

**Methods:**

Clinical, electrical, biological and psychological characteristics of the patient and his offspring were identified. Cytogenetic exploration using RHG banding and molecular screening of SCN5a gene mutations using High Resolution Melting and sequencing were carried out. Subsequently, genetic counselling was undertaken for all the family members and psychological concerns were reported.

**Results:**

A 51-year-old married man with an academic career was born from a consanguineous couple, with a family history of sudden cardiac death. He was diagnosed with BS1 based on the pathognomonic ST-segment elevation in leads V1–V3, after experiencing palpitations and syncope. He was treated by implantable cardioverter defibrillator. The patient was also being treated for diabetes and dyslipidemia. His children, a girl and two boys, were investigated by ECG, which revealed no electrical disorders. However, both boys reported chest pain on exertion. The 18-year-old girl presented with primary amenorrhea and infantilism, along with a Turner syndrome formula. Molecular analysis revealed none of the targeted mutations in the SCN5a gene. Psychologically, the patient had a phobia of death and reported painful sensations of imminent death at each palpitation. He was anxious about the clinical outcome of his children. The children reported anxiety about their autosomal dominant fathers’ disorder.

**Conclusions:**

Approximately 16% of BS patients experience depression and anxiety. More attention needs to be indorsed to the psychological distress of BG patients and their families.

**Disclosure of Interest:**

None Declared

